# Next generation sequencing to decipher concurrent loss of PMS2 and MSH6 in colorectal cancer

**DOI:** 10.1186/s13000-020-01001-2

**Published:** 2020-07-14

**Authors:** Esther Moreno, Juan M. Rosa-Rosa, Tamara Caniego-Casas, Ignacio Ruz-Caracuel, Cristian Perna, Carmen Guillén, José Palacios

**Affiliations:** 1 Department of Pathology, Hospital Universitario Ramón y Cajal, Carr de Colmenar Viejo, km. 9,100, 28034 Madrid, Spain; 2 CIBER-ONC Carlos III Health Institute, Madrid, Spain; 3 IRYCIS, Madrid, Spain; 4 Faculty of Medicine, University of Alcalá de Henares, Madrid, Spain; 5 Department of Medical Oncology, Hospital Universitario Ramón y Cajal, Madrid, Spain

**Keywords:** Colorectal cancer, Mismatch repair deficiency, Lynch syndrome, NGS, Case report

## Abstract

**Background:**

Immunohistochemistry (IHQ) is commonly used for the detection of mismatch repair proteins deficiency (MMRD). One very infrequent abnormal pattern of MMR protein expression is the loss of PMS2 and MSH6, with intact expression of MLH1 and MSH2.

**Case presentation:**

We review the frequency of this MMRD IHC pattern among 108 colorectal (CRCs) and 35 endometrial cancers in our files with loss of expression of at least one protein, and present two CRCs showing loss of PMS2 and MSH6 protein expression (1.9% of CRCs). NGS analysis of these tumours identified *PMS2* mutations (R134* germline mutation in one tumour and M1R and c.1239delA somatic mutation in the other) as the primary event and somatic *MSH6* mutation (c.3261dupC) as the secondary event in both tumours.

**Conclusions:**

This study suggests that Next Generation Sequencing (NGS) tumour analysis should be considered in the algorithm of Lynch syndrome screening to detect MMR gen somatic mutation in inconclusive cases.

## Background

Mismatch repair proteins deficiency (MMRD) can be studied by different methods in tumour tissue, but the most commonly used in the routine practice are immunohistochemistry (IHC) to analyze the expression of MMR proteins (MLH1, PMS2, MSH2 and MSH6) and/or microsatellite instability (MSI) analysis [1, 2]. Tumours with MMRD, in general, loss not only the expression of the protein coded by the altered gene, but also the paired protein in the heterodimer [3]. Since the most common molecular event leading to MMRD is *MLH1* promoter hypermetylation, the most common immunohistochemical pattern observed in MMRD tumours is MLH1/PMS2 loss [4]. This pattern also occurs in most cases of Lynch syndrome (LS) due to *MLH1* germline mutation, whereas MSH2/MSH6 loss occurs in LS due to *MSH2* germline mutations [3]. In addition to these two more common pattern of MMR protein expression in tumour with MMRD, other less frequent patterns, such as isolated losses of PMS2 and MSH6 also occurred in LS patients due to germline mutations of *PMS2* [1] and *MSH6* [5, 6], respectively.

One very infrequent abnormal pattern of MMR protein expression is the loss of PMS2 and MSH6, with intact expression of MLH1 and MSH2 [3]. In this study, we review the frequency of this IHC pattern among colorectal (CRCs) and endometrial cancers (ECs) in our files and present two CRCs showing loss of PMS2 and MSH6. Next Generation Sequencing (NGS) analysis identified *PMS2* mutations (germline in one tumour and somatic in the other) as the primary event and somatic *MSH6* mutation as the secondary event in both tumours. These cases illustrated the utility of NGS on tumour tissue for LS screening in inconclusive cases.

## Case presentation and results

### Case selection. Clinicopathological and molecular analysis

CRCs and ECs with loss of any MMR protein diagnosed between 2010 and 2019 were identified in our Laboratory Information System. Indications for the study of MMR protein expression have changed along this period of time from screening for LS in patients with CRC and Bethesda criteria to universal screening, not only for the identification of patients with LS, but also to select the more appropriate chemotherapy or immunotherapy treatment. In the same way, universal screening for LS by IHC has been implemented in our centre for all ECs. In two cases, IHQ of MMR protein and NGS were realized [7–9] [see Additional file 1].

### Clinicopathological features and immunohistochemistry

We identified 108 CRCs and 35 ECs with loss of at least one MMR protein. The different patterns of expression and their relative frequencies are presented in Table 1. Only 2 CRCs (1.9%) showed loss of PMS2 and MSH6 with intact expression of MLH1 and MSH2.

**Table 1:** Immunohistochemical patterns of MMR protein loss in colorectal and endometrial cancer

	MLH1/PMS2	MSH2/MSH6	PMS2	MSH6	4 MMR	MLH1/PMS2/MSH6	PMS2/MSH6	Total
**CRC**	85 / 78.7%	11 / 10.2%	3 / 2.8%	5 / 4.6%	1 / 0.9%	1 / 0.9%	2 / 1.9%	108 / 100%
**EC**	27 / 77.1%	3 / 8.6%	0 / 0%	5 / 14.3%	0 / 0%	0 / 0%	0%	35 / 100%

*CRC* colorectal cancer; *EC* endometrial cancer

**Fig. 1:**
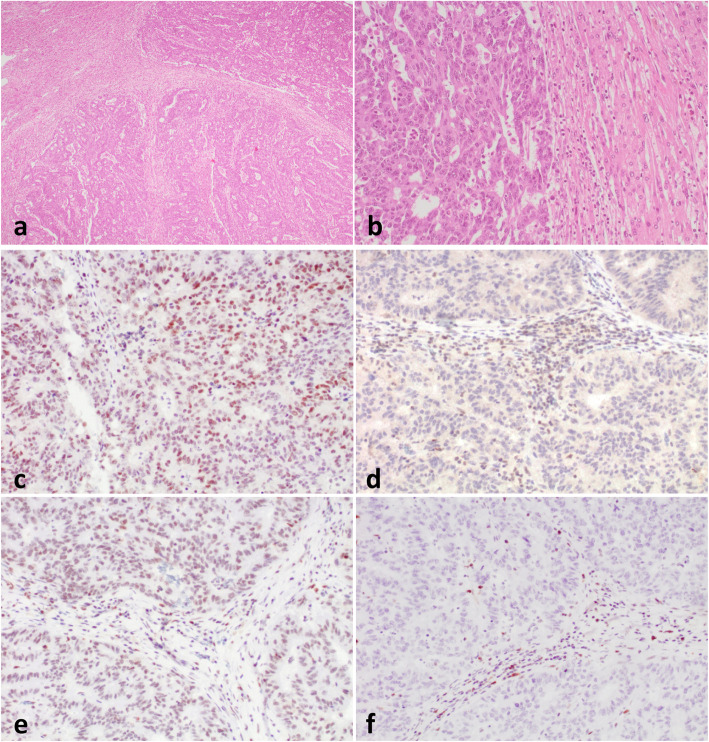
Liver metastases in patient 1. **a**-**b** Hematoxylin-eosin. **c** Preserved MLH1 staining. **d** Loss of PMS2 staining. **e** Intact expression of MSH2. **f** Loss of MSH6 expression

Patient 1 was a 41-year old female without a personal history of cancer, including father and grandfather with bladder cancer and a brother with melanoma. She presented in 2007 with a pT3N0 tumour of the right colon (a grade 2 adenocarcinoma of usual type without prominent lymphocytic infiltration) and received adjuvant Capecitabine. In 2016 a liver mass of 12 cm was identified and the patient received 3 cycles of neoadjuvant chemotherapy (FOLFOX). The pathologic examination showed the metastatic nature of the lesion and poor response to chemotherapy (approximately 10% of tumour regression). There was no evidence of disease in the last follow-up in 2020. Liver metastases showed loss of PMS2 and MSH6, but intact expression of MLH1 and MSH2 (Fig. 1). Immunostaining was then performed on the primary tumour, which showed isolated loss of PMS2.

Patient 2 was a 74-year old female without any personal or family history of disease. She presented in 2010 with a pT3N0 tumour of the transverse colon (a grade 2 usual adenocarcinoma with 20% mucinous differentiation and without prominent lymphocytic infiltration). The tumour showed loss of PMS2 and MSH6 staining with intact staining for MLH1 and MSH2 [see Additional file 2]. She received 12 cycles of adjuvant chemotherapy (FOLFOX) and there was no evidence of disease during the last follow-up in 2020.

### Molecular analysis

NGS was performed in the liver metastasis in patient 1 and in the primary tumour in patient 2. Poor DNA integrity precluded NGS analysis of the primary tumour in patient 1.

**Fig. 2:**
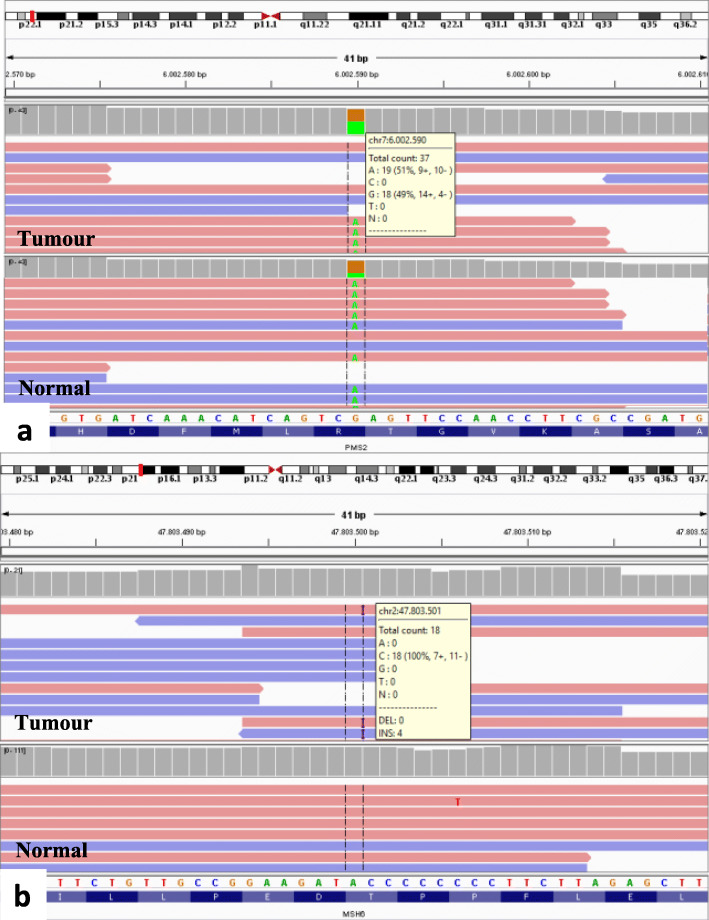
IGV view of *PMS2* and *MSH6* mutations in patient 1. **a** IGV view of *PMS2* R134* mutation in tumour and normal tissue. Variant allelic frequency (VAF) (green) was 50% in both samples. **b** IGV view of *MSH6* c.3261dupC mutation in tumour and normal tissue. VAF was 36% in tumour sample

The variants identified in tumour and normal tissue in both patients are shown in Table 2. In patient 1, a germline truncating mutation (R134*) was found in *PMS2*, accompanied by a somatic missense mutation (G271S). Patient 2 portrayed two putative truncating somatic mutations (M1R and c.1239delA) in *PMS2*. Interestingly, tumour samples from both patients portrayed the same somatic frameshift mutation in *MSH6* (c.3261dupC). In addition, a second missense somatic mutation located in a close genomic position to the previously one, was detected in both patients (R379I and R361H, respectively) (Fig. 2). Additional mutations are presented in Table 2. Mutations studied by Sanger sequencing were all confirmed [see additional file 1].

**Table 2:** Genetic variants detected by NGS in case 1 and case 2

Sample	Component	MLH1	PMS2	MSH6	POLE	TP53
**Case 1**	TT	K392T	G271S;R134*;**T485K**	R361H;c.3261dupC	E277G	
	NT	–	R134*;**T485K**	–	–	
**Case 2**	TT	E230*	M1R;c.1239delA	R379I;c.3261dupC	R446W	M246L
	NT	–	–	–	–	–

Germline variants are shown in bold. TT: Tumour tissue; NT: Normal tisue

## Discussion and conclusions

The concurrent loss of PMS2 and MSH6 expression with intact expression of MLH1 and MSH2 is a very infrequent IHQ pattern in human tumours [3]. In our review of 108 CRCs and 35 ECs with loss of any MMR protein, we detected only 2 cases among CRCs and no cases among ECs. In addition, we have recently reported the incidence of MMRD among 502 ovarian carcinomas. We detected MMRD in 31 endometrioid ovarian carcinomas and 3 clear cell carcinomas. Any of them showed the isolated loss of PMS2 and MSH6 [7].

Our data tend to support the hypothesis that in both tumours genetic alterations in *PMS2* produced MMRD and, secondarily, somatic mutations in *MSH6*, which induced inactivation of the gene and loss of the protein. In patient 2, two pathogenic somatic mutations were detected in *PMS2*, whereas in patient 1 we detected a pathogenic *PMS2* germline mutation, indicating LS, and a somatic variant of unknown significance. In both patients, *MSH6* carried the same somatic frameshift mutation (c.3261dupC) located in a mononucleotide tract, typical of MMRD.

Although a complete review of all published series about MMRD is out of the scope of this study, we have only identified two similar cases included in the series reported by McCarthy et al. [10], who found two CRCs with absent PMS2 and focal MSH6 expression in less than 2% of CRCs. Whereas one patient carried a pathogenic germline *PMS2* mutation, no *PMS2* alterations were reported in the other patient. The authors also observed a somatic mutation in the same mononucleotide tract in *MSH6* in three additional CRCs with MLH1/PMS2 loss, although the subjacent genetic or epigenetic alteration of these three cases was not reported.

Previous studies have reported that isolated loss of PMS2 represents approximately 0.4% of CRCs and 2.2–7% of ECs with MMRD, respectively [11, 12]. There are, however, few studies analysing the molecular alterations responsible of this IHC expression pattern. Approximately 60–70% of CRCs and ECs with isolated PMS2 seems to correspond to LS [11, 13]. Thus, Dudley et al. [11], observed that the main cause of isolated PMS2 loss was *PMS2* (47%) or *MLH1* (24%) germline mutation. These germline *MLH1* mutations produced MLH1 proteins that retain antigenicity but are non-functional compromising stability of MLH1-PMS2 complexes and inducing PMS2 loss. Kato et al. [2] reported 8 ECs with isolated PMS2 loss and observed that in 50% of these tumours the IHC alteration was due heterogeneous *MLH1* promoter hypermetylation. Only 1 out of 5 patients tested for germline mutations carried a *PMS2* germline mutation and any of them carried a *MLH1* germline mutations. Finally, Pearlman et al. [13] reported that 12–15% of tumours with isolated PMS2 carried a double somatic event in *PMS2*.

Tumours with isolated PMS2 loss seem to have specific clinicopathological features. The risk of developing CRC is lower among LS patients carrying germline *PMS2* mutations than among LS patients carrying germline mutations in other MMR genes (10–20% by age 70 years, instead of 40–50% with *MLH1* or *MSH2* mutations) [1]. In addition, CRCs with isolated loss of PMS2 expression showed a lower frequency of histologic features of MSI and a tendency toward aggressive behaviour, which may be related to less immune activation. Thus, the frequency of immune activation-related histologic features, such as increased TILs or Crohn-like lymphocytic infiltrates, was significantly lower in CRCs with isolated loss of PMS2 than in other MMR-deficient tumours [1]. In accordance with these observations none of the two tumours presented in this study had Crohn-like lymphocytic infiltrates or increased TILS. In addition, any of them was poorly differentiated or had medullary features.

In patient 1, MSH6 loss of expression occurred only in liver metastases and after the use of chemotherapy. MSH6 loss expression has been reported in some CRCs after the use of neoadjuvant therapy [14]. Although the chemotherapy treatment in our patient could have had some effect on MSH6 expression, the complete loss of expression was more probably related with the two detected somatic mutations.

These two cases showed a somatic mutation in the exonuclease domain of *POLE*. Whereas one of the mutations was reported as probably benign in previous reports, the somatic mutation E277G detected in the tumour of patient 1 has been reported as germline and pathogenic in one family with familial CRC [15]. The role of these mutations in cancer progression of these cases remains to be established.

Summarizing, loss of PMS2 and MSH6 expression with intact expression of MLH1 and MSH2 is very infrequent and was observed in 1.9% of CRC with IHC-MMRD, but not in ECs. NGS analysis of two cases demonstrated *PMS2* germline and/or somatic mutations as the possible primary event and somatic mutations of *MSH6* as a possible secondary event of these cases. NGS tumour analysis should be considered in the algorism of LS screening for inconclusive cases.

## Supplementary information

**Additional file 1.** Immunohistochemistry: Clones and criteria used for the interpretation. Massive parallel sequencing: Description of the NGS panel. Sanger sequencing: Primers used and variants obtained from Sanger sequencing.

**Additional file 2.** Figure case 2.

**Additional file 3.** 2013 CARE Checklist.

## Data Availability

Main data generated or analysed during this study are included in this published article and its supplementary information files [see Additional file 1].

## Abbreviations

IHQ: Immunohistochemistry
MMRD: Mismatch repair proteins deficiency
CRC: Colorectal cancer
EC: Endometrial cancer
NGS: Next Generation Sequencing
MSI: Microsatellite instability
LS: Lynch syndrome
VAF: Variant allelic frequency
