# Supplementary material for: Next generation sequencing to decipher concurrent loss of PMS2 and MSH6 in colorectal cancer
Source: Diagn Pathol. 2020 Jul 14;15:84. doi: 10.1186/s13000-020-01001-2 (PMC7362514; doi:10.1186/s13000-020-01001-2)
Supplement: Supplementary file 1 — Additional file 1. Immunohistochemistry: Clones and criteria used for the interpretation. Massive parallel sequencing: Description of the NGS panel. Sanger sequencing: Primers used and variants obtained from Sanger sequencing. [file 13000_2020_1001_MOESM1_ESM.docx]

**Immunohistochemistry**

In two cases with loss of PMS2 and MSH6, immunohistochemical stains were repeated for the study of the 4 MMR proteins. For immunohistochemistry, we used the following antibodies: MLH1 (clone ES05, Dako Glostrup, Denmark; prediluted), PMS2 (clone EP51, Dako, prediluted), MSH2 (clone FE11, Dako, prediluted) and MSH6 (clone EP49, Dako, prediluted). Staining was performed using OMNIS autostainer (Dako), as previously reported [1]. Loss of staining was defined as complete absence of nuclear staining in tumour cells with intact nuclear staining in normal cells, which served as positive control.

**Massive parallel sequencing**

Massive parallel sequencing was performed in both tumour and normal tissue using a custom panel of 47 genes frequently mutated in different types of cancers (including *MLH1*, *PMS2*, *MSH2*, and *MSH6*) and a MiSeq sequencing platform. The protocol for library preparation was already reported [2], and the bioinformatics analysis was detailed elsewhere [3].

**Sanger sequencing**

Sanger sequencing was performed to confirm dubious mutations. In case 1 the *POLE* mutation (E277G) was validated using the forward primer 5´ACCAGAGGGAGGTAGAGCAG 3´ and the reverse 5´GCTGCTGTAGTATGGGGACC 3´. In case 2 the *POLE* mutation (R446W) using the forward primer 5´GCTAGGCTATGATCCCGTGG 3´ and the reverse 5´TGGCCATCTGGATGCGTG 3´, the *PMS2* mutations (M1R) using the primer forward 5´ AAGCAGCCAATGGGAGTTCA 3´and the reverse 5´ GAGAGGGGACACCGGAAGA 3´, and the germline mutation in *BRCA2* (D189Y) with de forward primer 5´ CCAGGGTCGTCAGACACCAA 3´and reverse 5´ AGCACAGTAGAACTAAGGGTGG 3´.

| **Table 1** Variants obtained from Sanger sequencing of case 1 and case 2 | | | | | | |
| --- | --- | --- | --- | --- | --- | --- |
| **Sample** | **Variant** | **Gen** | **Chr position** | **Change** | **FW** | **RV** |
| **Case 1** | E277G | POLE | Chr12:132676625 | T>C | ACCAGAGGGAGGTAGAGCAG | GCTGCTGTAGTATGGGGACC |
| **Case 2** | M1R | PMS2 | Chr7:6009018 | A>C | AAGCAGCCAATGGGAGTTCA | GAGAGGGGACACCGGAAGA |
|  | D189Y | BRCA2 | Chr:32326547 | G>T | CCAGGGTCGTCAGACACCAA | AGCACAGTAGAACTAAGGGTGG |
|  | R446W | POLE | Chr12:132673598 | G>A | GCTAGGCTATGATCCCGTGG | TGGCCATCTGGATGCGTG |
| **Chr position** Chromosomal position. **FW** Forward primer. **RV** Reverse primer | | | | | | |


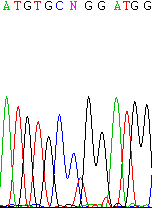

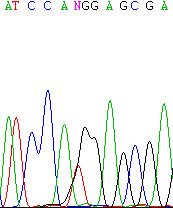


**a**

**b**


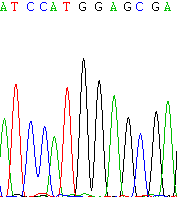


Tumour

Tumour

Normal

Normal


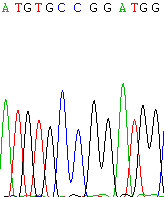


**Fig. 1** Electropherogram from Sanger sequencing in tumour and normal tissue of case 2. **a** Showing M1R *PMS2* mutation. **b** R446W *POLE* mutation

**REFERENCES**

1. Leskela S, Romero I, Cristobal E, et al (2020) Mismatch Repair Deficiency in Ovarian Carcinoma. Am J Surg Pathol 2020;44:649–656.
2. [Rosa-Rosa JM](https://www.ncbi.nlm.nih.gov/pubmed/?term=Rosa-Rosa%20JM%5BAuthor%5D&cauthor=true&cauthor_uid=30337841), [Caniego-Casas T](https://www.ncbi.nlm.nih.gov/pubmed/?term=Caniego-Casas%20T%5BAuthor%5D&cauthor=true&cauthor_uid=30337841), [Leskela S](https://www.ncbi.nlm.nih.gov/pubmed/?term=Leskela%20S%5BAuthor%5D&cauthor=true&cauthor_uid=30337841), et al (2018) Modified SureSelect(QXT) Target Enrichment Protocol for Illumina Multiplexed Sequencing of FFPE Samples. [Biol Proced Online.](https://www.ncbi.nlm.nih.gov/pubmed/?term=Modified+SureSelect(QXT)+Target+Enrichment+Protocol+for+Illumina+Multiplexed+Sequencing+of+FFPE+Samples+Biol+Proced+Online+20%3A19) 2018;20:19. <https://doi.org/10.1186/s12575-018-0084-7>
3. [Rosa-Rosa JM](https://www.ncbi.nlm.nih.gov/pubmed/?term=Rosa-Rosa%20JM%5BAuthor%5D&cauthor=true&cauthor_uid=30337841), [Caniego-Casas T](https://www.ncbi.nlm.nih.gov/pubmed/?term=Caniego-Casas%20T%5BAuthor%5D&cauthor=true&cauthor_uid=30337841), [Leskela S](https://www.ncbi.nlm.nih.gov/pubmed/?term=Leskela%20S%5BAuthor%5D&cauthor=true&cauthor_uid=30337841), et al (2019) High Frequency of ERBB2 Activating Mutations in Invasive Lobular Breast Carcinoma with Pleomorphic Features. [Cancers (Basel).](https://www.ncbi.nlm.nih.gov/pubmed/?term=High+Frequency+of+ERBB2+Activating+Mutations+in+Invasive+Lobular+Breast+Carcinoma+with+Pleomorphic+Features) 2019;11(1). <https://doi.org/10.3390/cancers11010074>
