# Supplementary material for: Next generation sequencing to decipher concurrent loss of PMS2 and MSH6 in colorectal cancer
Source: Diagn Pathol. 2020 Jul 14;15:84. doi: 10.1186/s13000-020-01001-2 (PMC7362514; doi:10.1186/s13000-020-01001-2)
Supplement: Supplementary file 2 — Additional file 2. Figure case 2. [file 13000_2020_1001_MOESM2_ESM.pptx]

## Slide 1
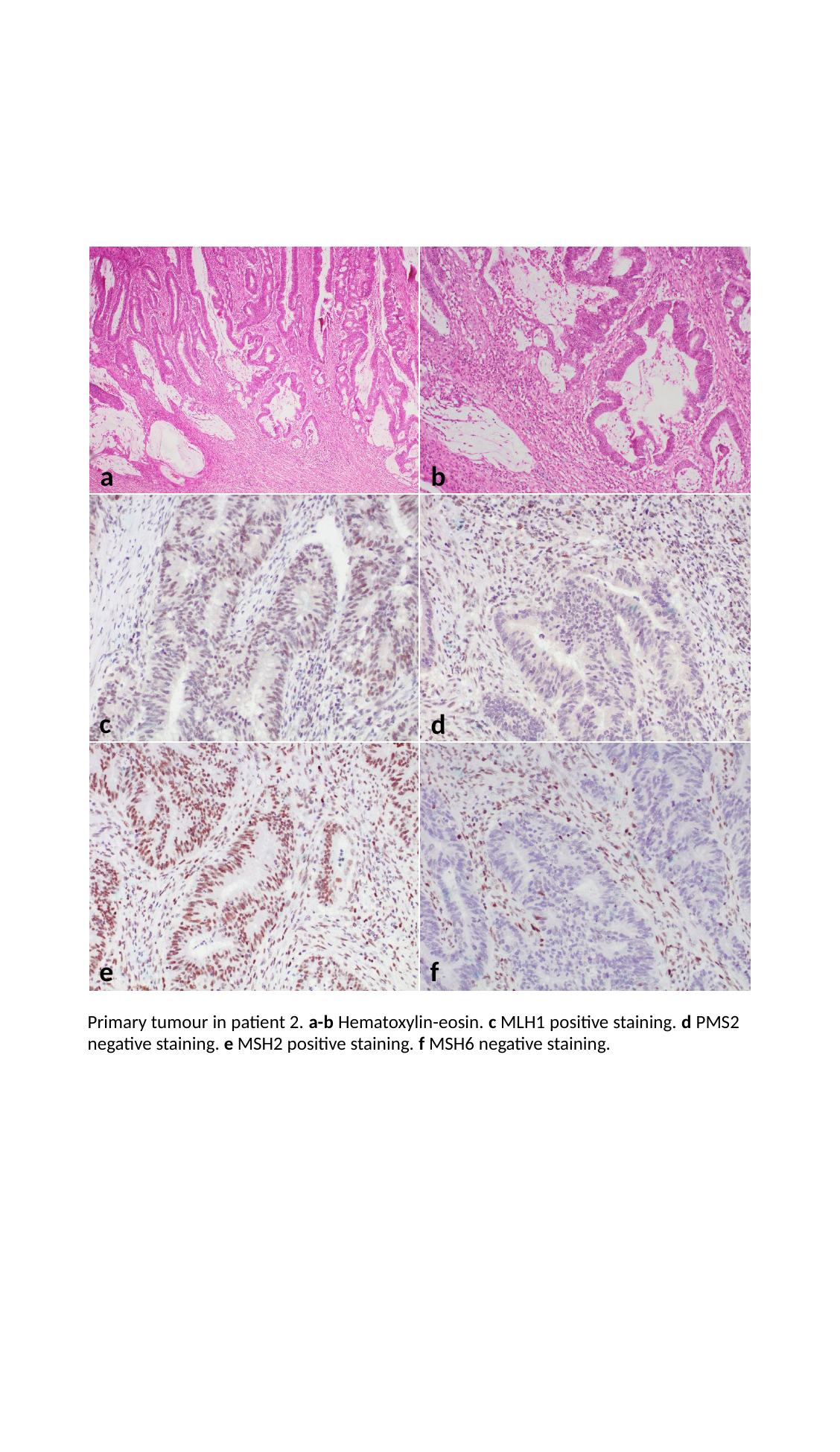

a
b
c
d
e
f
Primary tumour in patient 2. a-b Hematoxylin-eosin. c MLH1 positive staining. d PMS2 negative staining. e MSH2 positive staining. f MSH6 negative staining.
