# Supplementary material for: Next generation sequencing to decipher concurrent loss of PMS2 and MSH6 in colorectal cancer
Source: Diagn Pathol. 2020 Jul 14;15:84. doi: 10.1186/s13000-020-01001-2 (PMC7362514; doi:10.1186/s13000-020-01001-2)
Supplement: Supplementary file 3 — Additional file 3. 2013 CARE Checklist. [file 13000_2020_1001_MOESM3_ESM.docx]

**2013 CARE Checklist**

1. **Title** – The diagnosis or intervention of primary focus followed by the words “case report”……………………………………………………......................NO
2. **Key Words** – 2 to 5 key words that identify diagnoses or interventions in this case report (including "case report")…………...…..YES (page 2, line keywords)
3. **Abstract** – (structured or unstructured)
   - Introduction – What is unique about this case and what does it add to the scientific literature?..............................................................................YES
   - The patient’s main concerns and important clinical findings…………NO
   - The primary diagnoses, interventions, and outcomes…………………NO
   - Conclusion – What are one or more “take-away” lessons from this case report?..................................................................................................YES
4. **Introduction** – Briefly summarizes why this case is unique and may include medical literature references……..……YES (page 2, introduction - paragraph 2)
5. **Patient Information**
   - De-identified patient specific information...…….YES (case presentation)
   - Primary concerns and symptoms of the patient……………………….NO
   - Medical, family, and psychosocial history including relevant genetic information…………………………………………………………….NO
   - Relevant past interventions and their outcomes………………………NO
6. **Clinical Findings** – Describe significant physical examination (PE) and important clinical findings…………………………………………………….NO
7. **Timeline** – Historical and current information from this episode of care organized as a timeline (figure or table)………………………………………NO
8. **Diagnostic Assessment**
   - Diagnostic methods (PE, laboratory testing, imaging, surveys)……..YES (case presentation and additional file 1)
   - Diagnostic challenges………………………………………………..YES (case presentation)
   - Diagnosis (including other diagnoses considered)…………………..YES (case presentation)
   - Prognostic characteristics when applicable…………………………...NO
9. **Therapeutic Intervention**
   - Types of therapeutic intervention (pharmacologic, surgical, preventive)……………………………………...YES (case presentation)
   - Administration of therapeutic intervention (dosage, strength, duration)………………………………………...YES (case presentation)
   - Changes in therapeutic interventions with explanations………………NO
10. **Follow-up and Outcomes**
    - Clinician- and patient-assessed outcomes if available……………….YES (case presentation)
    - Important follow-up diagnostic and other test results………………..YES (case presentation)
    - Intervention adherence and tolerability. (How was this assessed?)…..NO
    - Adverse and unanticipated events…………………………………….NO
11. **Discussion**
    - Strengths and limitations in your approach to this case……………….NO
    - Discussion of the relevant medical literature…..…...YES (discussion and conclusion, all paragraphs)
    - The rationale for your conclusions……….…….…..YES (discussion and conclusion, paragraphs 5 & 6)
    - The primary “take-away” lessons from this case report (without references) in a one paragraph conclusion…….…...YES (discussion and conclusion, last paragraph
12. **Patient Perspective** – The patient should share their perspective on the treatment(s) they received……………………………………………………..NO
13. **Informed Consent** – The patient should give informed consent. (Provide if requested.)……………………………………………………………………YES
